# Pericardial Diseases Mortality Trends in Brazil From 2000 to 2022

**DOI:** 10.7759/cureus.57949

**Published:** 2024-04-10

**Authors:** Billy McBenedict, Yusuf A Ahmed, Reem Reda Elmahdi, Walaa H Yusuf, José Geraldo M Netto, Gabriella Valentim, Ana Abrahão, Bruno Lima Pessôa, Evandro T Mesquita

**Affiliations:** 1 Medicine, Antonio Pedro University Hospital, Niterói, BRA; 2 Medicine, Mansoura University, Mansoura, EGY; 3 Cardiology, Fluminense Federal University, Niterói, BRA; 4 Nursing, Fluminense Federal University, Niterói, BRA; 5 Public Health, Fluminense Federal University, Niterói, BRA; 6 Neurosurgery, Fluminense Federal University, Niterói, BRA; 7 Clinical Medicine, Antonio Pedro University Hospital, Níteroi, BRA

**Keywords:** joinpoint regression, mortality trends, pericardial effusion, pericarditis, pericardial diseases

## Abstract

Background

Pericardial diseases manifest in various clinical forms, including acute pericarditis, constrictive pericarditis, pericardial effusion, and cardiac tamponade, with acute pericarditis being the most prevalent. These conditions significantly contribute to mortality rates. Therefore, this article aimed to analyze mortality trends in the Brazilian population based on age and sex, shedding light on the impact of pericardial diseases on public health outcomes.

Methods

This is a retrospective time-series analysis of pericardial disease mortality rates in Brazil (2000-2022). Data was obtained from the Department of Informatics of the Unified Health System (DATASUS), and the 10th edition of the International Classification of Diseases (ICD-10) codes: I30, I31, and I32 were included for analysis. We gathered population and demographic data categorized by age range and sex from the Brazilian Institute of Geography and Statistics (IBGE). Subsequently, we computed the age-standardized mortality rate per 100,000 individuals and assessed the annual percentage changes (APCs) and average annual percentage changes (AAPCs) using joinpoint regression, along with their corresponding 95% confidence intervals (CIs).

Results

In terms of mortality trends based on sex, overall mortality rates remained stable for males and combined sexes over the study period. However, there was a notable increase in mortality rates among females (AAPC=1.18), particularly between 2020 and 2022, with a significant APC of 27.55. Analyzing pericardial diseases across different age groups (20 to 80 years and above), it wasobserved that mortality rates significantly increased in the 70-79 and 80 years and above age groups throughout the study period (AAPC=1.0339 and AAPC=3.4587, respectively). These two age groups experienced the highest significant rise in mortality between 2020 and 2022. Other age groups did not exhibit a significant change in AAPC.

Conclusions

This comprehensive analysis spanning two decades (2000-2022), examined the mortality trends of pericardial diseases in Brazil and revealed relative stability overall. Males exhibited an overall higher mortality number due to pericardial diseases; however, females showed the most significant increase in mortality trend throughout the whole period. In the first segment (2000-2015), mortality rose across all cohorts, which was attributed to substandard healthcare facilities and infectious diseases like tuberculosis. The second segment (2016-2020) saw a decline in mortality, likely due to improved healthcare, particularly the increased availability of echocardiograms. However, the third segment (2020-2022) witnessed a sharp rise in mortality, coinciding with the COVID-19 pandemic, with post-COVID-19 symptoms, particularly pericarditis. Pericarditis-related death rates declined compared to pericardial effusion, and mortality rates correlated directly with age, with older cohorts experiencing higher mortality due to increased comorbidities, and decline in health and immunocompetency.

## Introduction

Pericardial disorders encompass various pathologies such as acute pericarditis, constrictive pericarditis, pericardial effusion, and cardiac tamponade [1]. Among these, acute pericarditis emerges as the most prevalent condition affecting the pericardial sac [2]. Pericarditis manifests as an inflammation of the pericardial membrane and can be classified into acute, incessant, recurrent, or chronic forms [3]. Regarding sex and age patterns in pericarditis, a retrospective study based on data from 29 hospitals in Finland indicated that individuals aged 50-59 years faced a heightened risk of developing acute pericarditis, with males comprising the majority of cases [4]. Age plays a crucial role in the presentation of pericarditis, with younger patients typically exhibiting classic symptoms such as chest pain, pericardial rub, and characteristic electrocardiogram features like elevated ST segments and PR depressions. Conversely, older patients often present with dyspnea, elevated systolic blood pressure, and pericardial and pleural effusion, posing challenges in diagnosing pericarditis in this demographic [5].

Pericarditis, typically benign and self-limiting, can sometimes lead to complications like pericardial effusion or constriction, posing increased risks of morbidity and mortality. In developed nations, pericarditis accounts for approximately 0.1% of hospital admissions and is identified in 5% of patients presenting with non-cardiac chest pain in emergency departments [6]. The incidence of acute pericarditis varies globally, with studies reporting rates ranging from 5.7 per 100,000 persons per year in the United States to 10.1 cases per 100,000 persons per year in Switzerland [7,8]. Due to its often mild and self-resolving nature, mild cases of pericarditis may go undiagnosed, particularly in underdeveloped regions, leading to challenges in determining accurate incidence rates. However, untreated pericarditis can exacerbate and prolong recovery [9]. A Danish study comparing pericarditis patients to a matched-control group found a five-year survival rate of 92.9% in the pericarditis cohort, slightly lower than the 95.8% observed in the control group. Additionally, pericarditis patients had a higher admission rate for diagnosis and treatment [10].

In developed countries, constrictive pericarditis is primarily attributed to idiopathic factors, prior cardiac surgeries, and radiation exposure [11]. However, globally, tuberculosis (TB) remains a significant cause of pericarditis, particularly among individuals co-infected with human immunodeficiency virus (HIV) [6]. Low- and middle-income countries (LMICs), like Brazil, often see post-infectious constriction and TB as the predominant causes [12]. Notably, pericarditis of tuberculous origin is associated with higher perioperative fatality rates and substantial morbidity during pericardiectomy procedures [13,14]. Additionally, alongside HIV and acquired immunodeficiency syndrome (AIDS), and TB, arboviral infections such as dengue and Zika viruses are endemic in countries like Brazil [15]. The Brazilian Ministry of Health reported a significant increase in dengue cases in early 2024, indicating the ongoing threat posed by arboviral diseases [16].

Cardiac complications associated with dengue fever include atrioventricular conduction abnormalities, supra-ventricular arrhythmias, myocarditis, pericarditis, and myopericarditis [17]. While dengue pericarditis is infrequent, cases have been documented, often accompanied by pericardial effusions in severe instances [17]. Recent research involving approximately 500 participants aimed to assess the risk of developing constrictive pericarditis following an acute pericarditis diagnosis, aligning with the heightened burden of this condition in LMICs. The study identified tuberculous pericarditis (31.65 occurrences per 1000 patient-years) and purulent pericarditis (52.74 instances per 1000 patient-years) as the two conditions associated with the highest risk. However, purulent pericarditis appears to have become less common due to the widespread use of antibiotics and routine vaccination against previously prevalent bacterial pathogens [18].

Pericardial effusion refers to an abnormal accumulation of fluid within the pericardial sac [6]. This condition can manifest in various forms, ranging from asymptomatic fluid buildup incidentally detected through imaging to life-threatening scenarios associated with cardiac tamponade [19]. The causes of pericardial effusion are diverse and include idiopathic factors, malignancy, infectious agents, autoimmune conditions, uremia, hypothyroidism, pericardiotomy syndrome, and right-sided heart failure [6]. Demographic studies have shown varying trends regarding sex distribution, with some indicating a male predominance in pericardial effusion cases, while others report that it is significantly more common among women [20,21]. Furthermore, pericardial effusion affects individuals across all age groups, including pediatric, young adult, and elderly populations [20,22,23].

In a retrospective analysis, data from patients undergoing pericardiocentesis for pericardial effusion revealed that in-hospital mortality stood at 14.8%, while during follow-up (mean duration: 17.1 months), mortality escalated to 44.4% among these patients. Notably, in-hospital mortality was predominantly associated with idiopathic causes and hemodynamic instability, whereas medium-term follow-up mortality was primarily attributed to neoplastic origins [24]. Another recent investigation reported an in-hospital mortality rate of 5.67% among patients undergoing pericardiocentesis for pericardial effusion, with myocardial infarction-related myocardial rupture being the primary cause [23]. Additionally, even asymptomatic pericardial effusion in small volumes was correlated with heightened mortality [25].

A recent study conducted in Latin America, involving 106 patients with significant pericardial effusions, revealed that less than 10% of cases were of idiopathic origin, while infections accounted for less than 8% of cases, with TB being relatively rare [9]. In Brazil, idiopathic causes were identified as the primary culprit for pericardial effusion without tamponade, whereas post-surgical complications were the leading cause among inpatients with pericardial effusion accompanied by cardiac tamponade. The overall mortality rate in the Brazilian study was 31.5%, with higher mortality observed in patients presenting with cardiac tamponade concurrent with pericardial effusion [26].

Brazil, the largest country in South America and one of the most densely populated nations, is geographically divided into five regions: North, Northeast, Central-West, Southeast, and South [27]. Our study focuses on adult mortality rates due to pericarditis and pericardial effusion, encompassing both sexes of Brazil. Despite significant advancements in the Brazilian healthcare system over the past decade, marked by an increase in the number of hospitals and healthcare providers, there remains a research gap necessitating comprehensive investigations into the rising mortality rates associated with non-ischemic causes of chest pain, particularly, pericarditis and pericardial effusion [28]. Notably, existing literature lacks studies detailing the mortality rates of pericarditis and pericardial effusion in Brazil. 

Hence, the principal aim of this article was to meticulously scrutinize and contrast the patterns of mortality associated with pericardial diseases in Brazil. The overarching goal was to conduct a comprehensive analysis of mortality data related to pericardial diseases, thereby presenting a holistic global perspective. Furthermore, this involved a detailed examination of mortality rates, which attributed to specific manifestations of pericardial diseases, namely pericarditis and pericardial effusion, within the context of Brazil from 2000 to 2022, with a specific focus on trends concerning age and sex demographics. This in-depth analysis not only sheds light on the overall burden of pericardial diseases but also provides valuable insights into the nuances of mortality patterns, enabling a deeper understanding of the epidemiological landscape and potentially informing targeted interventions and healthcare strategies.

## Materials and methods

Study design and data collection

We conducted a descriptive time-series analysis using mortality data for pericarditis and other pericardial diseases in Brazil spanning from 2000 to 2022. The mortality data was sourced from the Department of Informatics of the Unified Health System (DATASUS) of the Brazilian Ministry of Health. DATASUS, operating under Brazil's Unified Health System (Sistema Único de Saúde or SUS), compiles information from hospitalizations reimbursed by SUS, covering approximately 80% of the Brazilian population. Records were identified using the 10th edition of the International Classification of Diseases (ICD-10). Specifically, mortality data for diseases coded as I30 (acute pericarditis), I31 (other diseases of the pericardium), and I32 (pericarditis in diseases classified elsewhere) between 2000 and 2022 were included, with analysis performed based on age groups and sex.

Population estimates and mortality rates

We acquired population and demographic data categorized by age groups and sex from the population estimates provided by the Brazilian Institute of Geography and Statistics (IBGE), available under the Demographic and Socioeconomic Information section. The data were organized using Google Sheets (Google, Inc., Mountain View, US), and percentages were computed based on sex and disease categories. We determined the age-standardized mortality rate for pericarditis and other pericardial diseases across all age groups. Mortality rates were expressed per 100,000 individuals, and age-adjusted rates were derived through direct standardization using the world standard population.

Time-series analysis

To analyze the temporal trends in mortality rates related to pericarditis and other pericardial diseases, we utilized joinpoint regression to calculate the average annual percentage changes (AAPCs) along with their corresponding 95% confidence intervals (CIs). The AAPC was computed as a geometrically weighted mean of the annual percentage change (APC) values obtained from the regression analysis. The trend analysis was conducted using the Joinpoint Regression Program (Version 5.0.2. May 2023), developed by the Statistical Research and Applications Branch of the National Cancer Institute (Bethesda, MD, United States). Weighted Bayesian Information Criteria were employed to determine the significance levels and identify the optimal combination of line segments and joinpoints. This analysis aimed to explore variations in mortality trends associated with pericarditis and other pericardial diseases across different age groups and sexes.

## Results

Sex and disease groups

In our study, we found that pericarditis (coded as I30 and I32) accounted for 857 female deaths and 1241 male deaths. Moreover, other diseases of the pericardium (coded as I31) resulted in 5643 fatalities among females and 6690 among males. When considering all pericardial diseases (I30, I31, and I32), our analysis revealed a total of 6500 female deaths and 7931 male deaths. Deaths attributed to pericarditis comprised 14.54% of all mortality related to pericardial diseases, with males representing 59.15% and females 40.85% of these cases. The remaining 85.46% of mortality was associated with other diseases of the pericardium, with 56.67% occurring in men and 45.75% in women. Overall, male deaths from pericardial diseases accounted for 54.95% of the total, while female deaths constituted the remaining 45.04%. These findings underscore the need for further exploration of sex-specific prevalence patterns of pericardial diseases within the Brazilian population.

Pericardial diseases

Pericardial Diseases Mortality Rates Based on Sex

**Figure 1:**
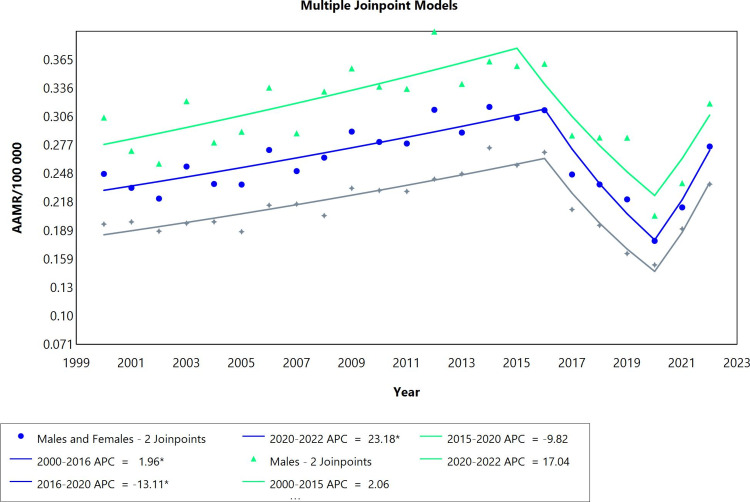
Pericardial diseases AAMR trend joinpoint analysis for the variable sex during the period of 2000-2022. *Significant at P<0.05 level AAMR: age-adjusted mortality rate; APC: annual percent change

The pericardial diseases mortality rate trend for both males and females combined, as well as the distinct males’ group, was stable overall throughout 2000 to 2022, unlike the female group in which the overall changes during this period were significant. All three categories had three segments each with two join points (Figure 1). Annual percent changes per segment for the combined sexes as well as the females’ group only were noteworthy. In addition, the rise in mortality was more pronounced among females than males (see AAPC values). Starting, there was an increase during the first period (2000 to 2016), followed by a significant decreasing trend with an APC value of -13.11 from 2016 to 2020 for the combined sexes group. The final period of the study for this group witnessed the highest mortality rates due to pericardial diseases (APC=23.18; CI: 8.34 to 36.64).* *

Examining the APC values of males, it is notable that the three segments exhibited fluctuations similar to those observed in the combined group of males and females. However, neither of these fluctuations in the trend were significant mortality rate changes. In the analysis of the females’ group, the changes in mortality rates were also similar to the combined group of males and females and it was distinguished by being the most pronounced throughout the whole period (AAPC=1.18; CI: 0.69 to 1.59). Although the females group recorded the most notable decline in mortality rates in the period 2016-2020 (APC=-13.62; CI: -17.85 to -10.14), the most prominent upward surge among all three categories happened to fall in the period spanning from 2020 to 2022 in the females group (APC=27.55; CI: 18.42 to 35.71) (Table 1).

**Table 1:** Trends in mortality related for pericardial diseases from 2000 to 2022. *Significant at P<0.05 level AAPC: average annual percent change; APC: annual percent change

Sex	Period	APC (95% CI)	AAPC (95% CI)
Males and females	2000-2016	1.96* (1.29 to 2.80)	0.75 (-0.18 to 1.33)
Males and females	2016-2020	-13.11* (-20.94 to -8.77)	
Males and females	2020-2022	23.18* (8.34 to 36.64)	
Males	2000-2015	2.06 (-1.98 to 3.80)	0.47 (-1.04 to 1.34)
Males	2015-2020	-9.82 (-21.12 to 7.21)	
Males	2020-2022	17.04 (-5.18 to 33.00)	
Females	2000-2016	2.25* (1.80 to 2.78)	1.18* (0.69 to 1.59)
Females	2016-2020	-13.62* (-17.85 to -10.14)	
Females	2020-2022	27.55* (18.42 to 35.71)	

Pericardial Diseases Mortality Rates Based on Age

**Figure 2:**
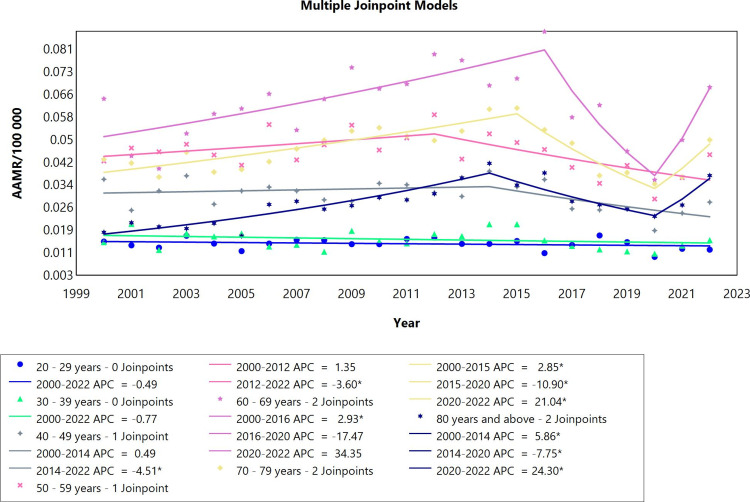
Pericardial diseases AAMR trend joinpoint analysis for the variable age during the period of 2000-2022. *Significant at P<0.05 level AAMR: age-adjusted mortality rate; APC: annual percent change

In the analysis of pericardial diseases by age groups in adults (males and females combined) ranging from 20 years to above 80 years in the period 2000-2022, the data was divided into seven cohorts (Figure 2, Table 2). The first two groups, the 20-29- and 30-39-year-old categories, manifested a stable mortality rate throughout the whole period. The clusters of 40-49 and 50-59-year-olds each had one joinpoint. For the first phase, the mortality rates remained stable for the two groups, followed by a significant decrease in the remaining period of the study with APC values of -4.51 and -3.60, respectively. For individuals aged 60-69 years, the mortality rates initially rose significantly with an APC of 2.93 in the period 2000 to 2016, and then no statistically significant changes in mortality trends were noted. Moreover, 70-79 and 80 years and above cohorts showed similar fluctuating patterns of mortality rates starting with a rise, then a decrease, followed again by a much steeper increase in rates between 2020 and 2022, which were all significant. During this period particularly, it is noteworthy to mention the most significant surge in mortality rates was among the 80 years and above patients (APC=24.30 CI: 4.38 to 40.32), followed by the second highest upward trend for the 70-79 age group (APC=21.04 CI:7.34 to 32.40). Overall mortality trends did not exhibit a significant change for all age groups except for the age groups 70-79 and 80 years and above with AAPC values of 1.03 and 3.46, respectively.

**Table 2:** Trends in mortality related to pericardial diseases among Brazilians aged 20 years or older from 2000 to 2022. *Significant at P<0.05 level AAPC: average annual percent change; APC: annual percent change

Age range	Period	APC (95% CI)	AAPC (95% CI)
20-29 years	2000-2022	-0.49 (-1.50 to 0.49)	-0.49 (-1.50 to 0.49)
30-39 years	2000-2022	-0.77 (-2.43 to 0.85)	-0.77 (-2.43 to 0.85)
40-49 years	2000-2014	0.49 (-1.54 to 18.06)	-1.36 (-3.42 to 0.86)
40-49 years	2014-2022	-4.51* (-21.05 to -0.74)	
50-59 years	2000-2012	1.35 (-0.25 to 10.32)	-0.93 (-2.19 to 0.42)
50-59 years	2012-2022	-3.60* (-11.71 to -1.46)	
60-69 years	2000-2016	2.93* (1.26 to 5.02)	1.30 (-0.70 to 2.53)
60-69 years	2016-2020	-17.47 (-31.42 to 2.16)	
60-69 years	2020-2022	34.35 (-3.67 to 68.21)	
70-79 years	2000-2015	2.85* (2.08 to 3.78)	1.03* (0.33 to 1.64)
70-79 years	2015-2020	-10.90* (-17.62 to -7.68)	
70-79 years	2020-2022	21.04* (7.34 to 32.40)	
80 years and above	2000-2014	5.86* (4.41 to 7.79)	3.46* (2.33 to 4.48)
80 years and above	2014-2020	-7.75* (-17.81 to -3.97)	
80 years and above	2020-2022	24.30* (4.38 to 40.32)	

Pericarditis

Pericarditis Mortality Rates Based on Sex

**Figure 3:**
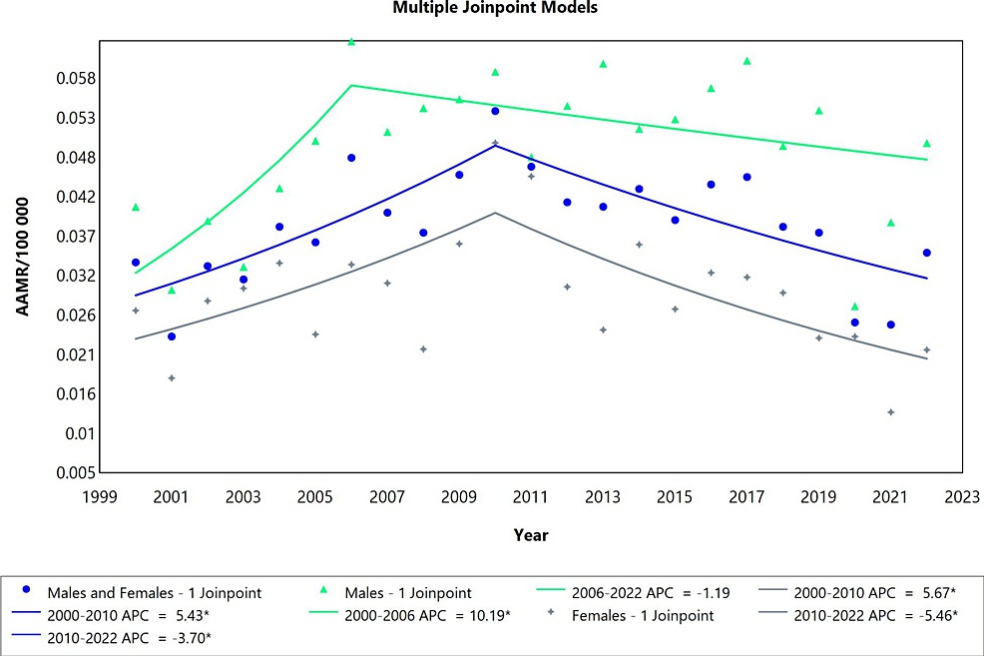
Pericarditis AAMR trend joinpoint analysis for the variable sex during the period of 2000-2022. *Significant at P<0.05 level AAMR: age-adjusted mortality rate; APC: annual percent change

The mortality trend for pericarditis showed one joinpoint in all studied groups (males, females, and combined males and females) (Figure 3). There was a significant rise in mortality for both sexes from 2000 to 2010 (APC=5.43; CI: 2.38 to 12.63) followed by a decrease in mortality from 2010 to 2022 (APC=-3.70; CI: -8.09 to -1.55). In females, the mortality followed a similar trend as it increased initially from 2000 to 2010 (APC=5.67; CI: 1.74 to 16.60) then it decreased from 2010 to 2022 (APC=-5.46; CI: -12.19 to -2.70). In males, the trend of mortality was slightly different as it increased from 2000 to 2006 (APC=10.19; CI: 2.94 to 40.50) then it remained stable from 2006 to 2022 (APC=-1.19; CI: -5.09 to 0.36). In all groups, there were no statistically significant changes in AAPC (Table 3).* *

**Table 3:** Trends in mortality for pericarditis from 2000 to 2022. *Significant at P<0.05 level AAPC: average annual percent change; APC: annual percent change

Sex	Period	APC (95% CI)	AAPC (95% CI)
Males and females	2000-2010	5.43* (2.38 to 12.63)	0.35 (-1.21 to 1.99)
Males and females	2010-2022	-3.70* (-8.09 to -1.55)	
Males	2000-2006	10.19* (2.94 to 40.50)	1.79 (-0.07 to 4.17)
Males	2006-2022	-1.19 (-5.08 to 0.36)	
Females	2000-2010	5.67* (1.74 to 16.60)	-0.56 (-2.78 to 1.59)
Females	2010-2022	-5.46* (-12.19 to -2.70)	

Pericarditis Mortality Rates Based on Sex

**Figure 4:**
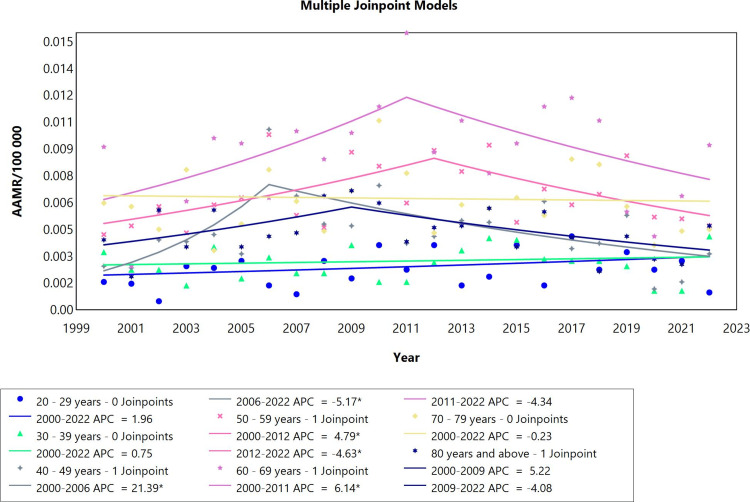
Pericarditis AAMR trend joinpoint analysis for the variable age during the period 2000-2022. *Significant at P<0.05 level AAMR: age-adjusted mortality rate; APC: annual percent change

The data for pericarditis was also analyzed for all age groups from 20 years to 80 years and above for the study period 2000-2022 (Figure 4, Table 4). Both age groups, 20-29 years and 30-39, years did not show significant changes in AAPC throughout the whole period. The 40-49- and 50-59-years-old categories showed one joinpoint and two segments. The first segment of both groups showed a significant upward shift, and the second segment exhibited a significant downward shift in mortality rates in the study period. One notable difference between both groups is that the joinpoint for the age groups 40-49 and 50-59 were recorded in 2006 and 2012, respectively. The category of adults aged 60-69 years showed one joinpoint that was preceded by an upward shift in mortality rates in 2000-2011 (APC=6.14; CI: 0.87 to 58.01) followed by a stability of the trend in 2011-2022. The cohort of adults aged 70-79 and 80-89 did not show any joinpoints throughout the whole period. The AAPC remained the same across all age groups.

**Table 4:** Trends in mortality for pericarditis among Brazilians aged 20 years or older from 2000 to 2022. *Significant at P<0.05 level AAPC: average annual percent change; APC: annual percent change

Age	Period	APC (95% CI)	AAPC (95% CI)
20-29 years	2000-2022	1.96 (-1.99 to 6.58)	1.96 (-1.99 to 6.58)
30-39 years	2000-2022	0.75 (-2.28 to 4.03)	0.76 (-2.28 to 4.03)
40-49 years	2000-2006	21.39* (7.43 to 81.72)	1.44 (-2.01 to 5.78)
40-49 years	2006-2022	-5.17* (-10.97 to -2.36)	
50-59 years	2000-2012	4.79* (1.51 to 24.45)	0.40 (-1.99 to 3.01)
50-59 years	2012-2022	-4.63* (-19.97 to -0.56)	
60-69 years	2000-2011	6.14* (0.87 to 58.01)	0.76 (-4.11 to 6.61)
60-69 years	2011-2022	-4.34 (-32.47 to 0.47)	
70-79 years	2000-2022	-0.23 (-2.78 to 2.37)	-0.23 (-2.78 to 2.37)
80 years and above	2000-2009	5.22 (-1.50 to 66.31)	-0.38 (-4.83 to 4.91)
80 years and above	2009-2022	-4.08 (-29.44 to 0.16)	

Pericardial effusion

Pericardial Effusion Mortality Rates Based on Sex

**Figure 5:**
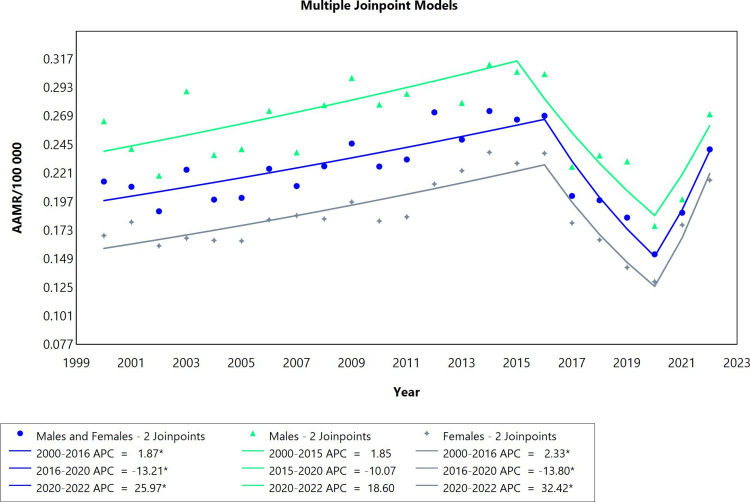
Pericardial effusion AAMR trend joinpoint analysis for the variable sex during the period 2000-2022. *Significant at P<0.05 level AAMR: age-adjusted mortality rate; APC: annual percent change

Pericardial effusion mortality rates in Brazil were represented by three cohorts based on sex (males, females, combined males and females groups). the presence of two joinpoints was common among the three cohort groups (Figure 5). There was a significant increase in mortality for both sexes from 2000 to 2016 (APC=1.87; CI: 1.15 to 2.78) followed by a decline in mortality rates from 2016 to 2020 (APC=-13.21; CI: -21.47 to -8.45) and eventually the mortality rose from 2020 to 2022 (APC=25.97; CI: 9.69 to 40.78). In females, the mortality followed a similar trend as it increased initially from 2000 to 2016 (APC=2.33; CI: 1.67 to 3.15). In 2016 to 2020 mortality dropped (APC=-13.80; CI: -21.14 to -9.43) then rose again from 2020-2022 (APC=32.42; CI:17.53 to 46.12). The mortality trend in males followed a similar pattern; however, it was not significant throughout the whole period (Table 5). The only group that exhibited an overall significant rise over the whole period from 2000 to 2022 (AAPC=1.54; CI: 0.77 to 2.13) was the females group.

**Table 5:** Trends in mortality related to pericardial effusion among Brazilians from 2000 to 2022. *Significant at P<0.05 level AAPC: average annual percent change; APC: annual percent change

Sex	Period	APC (95% CI)	AAPC (95% CI)
Males and females	2000-2016	1.87* (1.15 to 2.78)	0.87 (-0.07 to 1.50)
Males and females	2016-2020	-13.21* (-21.47 to -8.50)	
Males and females	2020-2022	25.97* (9.69 to 40.78)	
Males	2000-2015	1.85 (-5.61 to 4.51)	0.39 (-1.45 to 1.44)
Males	2015-2020	-10.07 (-23.81 to 12.69)	
Males	2020-2022	18.60 (-6.23 to 40.48)	
Females	2000-2016	2.33* (1.67 to 3.15)	1.54* (0.77 to 2.13)
Females	2016-2020	-13.80* (-21.14 to -9.43)	
Females	2020-2022	32.42*(17.53 to 46.12)	

Pericardial Effusion Mortality Rates Based on Age

**Figure 6:**
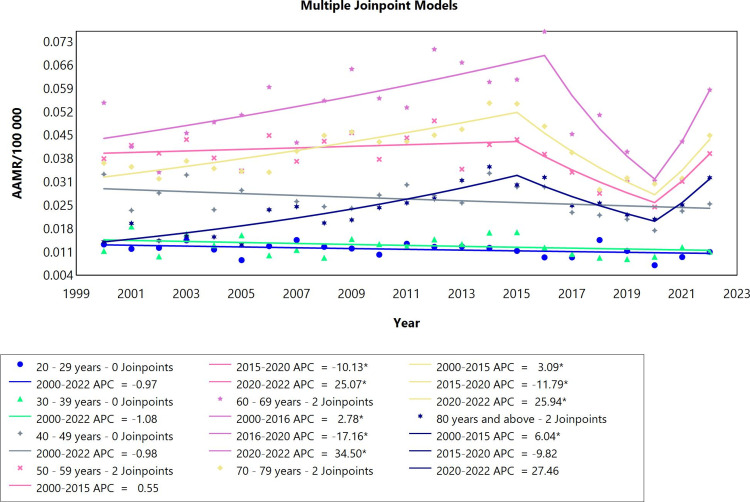
Pericardial effusion AAMR trend joinpoint analysis for the variable age during the period 2000-2022. *Significant at P < 0.05 level AAMR = age-adjusted mortality rate; APC = annual percent change

In the age groups spanning from 20 to 49 years, there were no statistically significant changes in pericardial effusion mortality rates in the whole study period (Table 6). In the category of adults aged 50-59, there were two joinpoints (Figure 6). In the period 2000-2015, the mortality rate did not exhibit a statistically significant change. It dropped significantly in the period 2015 to 2020 (APC=-10.13) and rose again in the period 2020-2022 (APC=25.07). The mortality trends in the age group 60-69 years had a fluctuating pattern. It initially increased in the period 2000-2016 (APC=2.78; CI: 1.43 to 4.60), dropped in the period 2016 to 2020 (APC=-17.16; CI: -29.52 to -8.29), followed by an increase in the period 2020 to 2022 (APC=34.50; CI: 4.97 to 63.29). The age group 70-79 had the same fluctuation and number of joinpoints as the age group 60-69 except that the first joinpoint was in 2015 rather than 2016. In the 80 years and above group, there were two joinpoints. From 2000 to 2015, there was a statistically significant increase (APC=6.04; CI: 3.94 to 8.68), followed by a non-significant decrease (ACP=-9.82; CI: -22.79 to 7.11) and increase (APC=27.46; CI: -1.82 to 50.51) in the periods 2015-2020 and 2020-2022, respectively. The overall mortality in the whole period showed a statistically significant increase in the age groups 70-79 (AAPC=1.33; CI: 0.11 to 2.16), and 80 years and above (AAPC=3.93; CI: 2.28 to 5.28).

**Table 6:** Joinpoint analysis of the trend in AAMR for pericardial effusion across different age groups from 2000 to 2022. *Significant at P < 0.05 level AAPC: average annual percent change; APC: annual percent change

Age	Period	APC (95% CI)	AAPC (95% CI)
20-29 years	2000-2022	-0.97 (-2.06 to 0.08)	-0.97 (-2.06 to 0.08)
30-39 years	2000-2022	-1.08 (-3.10 to 0.88)	-1.08 (-3.10 to 0.88)
40-49 years	2000-2022	-0.98 (-2.21 to 0.21)	-0.98 (-2.22 to 0.21)
50-59 years	2000-2015	0.55 (-0.57 to 2.05)	-0.02 (-1.25 to 0.84)
50-59 years	2015-2020	-10.13* (-20.76 to -4.82)	
50-59 years	2020-2022	25.07* (2.59 to 44.26)	
60-69 years	2000-2016	2.78* (1.43 to 4.56)	1.27 (-0.23 to 2.46)
60-69 years	2016-2020	-17.16* (-29.52 to -8.29)	
60-69 years	2020-2022	34.50* (4.97 to 63.29)	
70-79 years	2000-2015	3.09* (2.01 to 4.49)	1.33* (0.10 to 2.16)
70-79 years	2015-2020	-11.79* (-22.87 to -7.34)	
70-79 years	2020-2022	25.94* (5.08 to 43.23)	
80 years and above	2000-2015	6.04* (3.94 to 8.68)	3.93* (2.28 to 5.28)
80 years and above	2015-2020	-9.82 (-22.79 to 7.11)	
80 years and above	2020-2022	27.46 (-1.82 to 50.51)	

## Discussion

Pathophysiology of pericarditis

The pericardium plays a vital role in enhancing the dynamic interaction between the cardiac chambers, serving as a protective barrier against extrinsic infections, and providing structural support by anchoring the heart within the thoracic cavity [29]. Pathological responses of the pericardium to injury are characterized by intense inflammation, often accompanied by effusion, leading to the clinical manifestation of pericarditis. Pericarditis can stem from various etiologies including viral, bacterial, fungal, and uremic agents; post-acute myocardial infarction; neoplasms, cardiac surgical procedures, mediastinal irradiation; and systemic autoimmune disorders [30].

The pericardium is susceptible to a range of infectious agents, of which the most frequent culprits are bacteria of the genus *Staphylococcus*, *Streptococcus*, *Haemophilus*, and *Mycobacterium tuberculosis (MTb),* and viruses (coxsackie viruses, influenza virus, enteric cytopathogenic human orphan virus, and, more recently, COVID-19). While viral pericarditis often resolves without leaving fibrous sequelae, suggesting a self-limiting nature, effusions frequently occur when lymphocytes dominate the inflammatory response to viral antigens [6]. Studies have shown that viral pericarditis is associated with elevated serum cardiac troponin I (cTnI) levels, which correlate with the presence of pericardial effusion and ST-segment elevation. Despite this elevation in cTnI levels, it does not significantly affect prognosis or the development of complications such as tamponade and fibrosis, although it may be more pronounced in the presence of heightened myocardial inflammation [31].

A study reported that approximately 70% of cases involving significant pericardial effusion in developing countries like Brazil are linked to TB and HIV co-infection [29]. MTb can reach the pericardium through retrograde lymphatic or hematogenous dissemination from initial sites of infection [30]. With a high abundance of mycobacteria, an early fibrinous exudate with leukocytosis and early granuloma development occurs, followed by a sero-sanguineous effusion that primarily contains lymphocytes. As the granulomatous structure forms to contain the mycobacterial spread, the effusion gradually resolves. Deposition of collagen, fibrin, and extracellular matrix (ECM) leads to pericardial thickening and fibrosis [30].

The rich innervation of the parietal layer of the pericardium renders it sensitive to inflammation, often leading to pronounced retrosternal chest discomfort, a hallmark symptom of acute pericarditis [31]. This heightened sensitivity elucidates why chest discomfort is prevalent in over 90% of cases [12]. As fluid accumulates gradually in pericardial effusion, the compliance of the pericardium may increase, allowing the pericardial sac to expand without compressing the heart chambers [32]. Consequently, the rate of fluid accumulation, which impacts pericardial compliance and resultant pressure changes, often holds greater significance than the volume itself in determining hemodynamic consequences for the heart. The primary long-term complications of pericarditis include constrictive pericarditis and death, while short-term complications may involve cardiac tamponade with or without hemodynamic instability [32]. Chronic inflammation, thickening, adhesion, fibrosis, and eventual obliteration of the pericardial space are typical outcomes leading to the latter.

Pericardial diseases mortality rates based on sex and age

Our findings revealed a higher mortality rate among males compared to females throughout the entire study period, delineated across three distinct segments. Research indicates that men exhibit a twofold incidence rate of acute pericarditis compared to women [4]. Conversely, females have been linked to complications arising from acute pericarditis; however, this association was not found to be an independent factor influencing mortality rates [6]. Experimental studies have suggested that testosterone may promote a type of inflammation implicated in fibrosis within the context of myocarditis [32]. Pericarditis and myocarditis are more prevalent among younger males, with sex hormones, particularly testosterone, appearing to contribute to the differential susceptibility of pericarditis between sexes [3]. The initial segment spanning from 2000 to 2015 witnessed an increase in mortality rates across all three cohorts, potentially attributable to limited access to healthcare facilities and subpar healthcare services, particularly prevalent in LMICs like Brazil.

Cardiovascular disease tops the list of preventable deaths within the healthcare system, accounting for approximately 33% (2,817,000) of all fatalities, with 84% (2,358,000) attributed to the provision of inadequate healthcare services [33]. In LMICs, preventable diseases contribute to 8.6 million deaths, of which five million are linked to poor-quality healthcare received by patients who access the system. This figure surpasses the mortality rates from diabetes by over threefold and exceeds the global death toll from HIV/AIDS by fivefold. Within these nations, approximately 58% of avoidable deaths are associated with substandard medical care [33]. Between 2000 and 2015, Brazil experienced a surge in various infectious diseases. TB and post-infectious constriction emerge as primary contributors to pericarditis in LMICs like Brazil [12]. This is particularly significant as pericardial diseases of tuberculous origin are linked to heightened perioperative fatality rates and considerable morbidity [13,14]. A study found that the overall mortality ratio in presumed tuberculous pericarditis patients was 26% for a period of six months since being diagnosed [34]. However, patients who had HIV clinical signs showed a higher mortality ratio in comparison to patients without HIV clinical signs as they reached 40% and 17% mortality rates respectively. Death was also noted to be higher when there was a coexistence of pulmonary TB alongside being older in age.

In addition to HIV/AIDS and TB, arboviral infectious diseases, including dengue and Zika viruses, are endemic in several countries, including Brazil [15]. Cardiac complications observed in dengue patients encompass irregularities in atrioventricular conduction, supraventricular arrhythmias, myocarditis, pericarditis, and even myopericarditis. While rare, instances of dengue pericarditis have been documented. Although pericardial effusions are relatively uncommon, they have been reported in severe cases of dengue [17]. Bacterial pericarditis is commonly caused by Staphylococcus, Streptococcus, Haemophilus, and MTb infections. Patients who develop and are treated for purulent pericarditis may record high mortality rates reaching 40% [35]. The causes of death may be a result of the development of cardiac tamponade, decompensation, and constriction alongside systemic dissemination of the infection [35]. Although pericarditis due to methicillin-resistant Staphylococcus aureus (MRSA) is extremely rare in the antimicrobial era, it carries a mortality rate of up to 60% which is due to the development of complications, primarily cardiac tamponade [36]. 

Autoimmune diseases have a significant contribution to the mortality rates due to pericardial diseases; a study focused on systemic lupus erythematosus (SLE)found that patients who had lupus pericarditis had a significantly lower survival rate compared to patients having SLE without pericarditis in a follow-up period of 10 years, as patients with lupus pericarditis had a survival rate of 62.7%. In contrast, patients without pericarditis had a survival rate of 80.7%. Moreover, even if cardiac tamponade was absent in pericarditis patients, survival rates were significantly affected. Furthermore, patients with SLE having lupus pericarditis were noted to have higher disease activity, lower C3 levels, and increased comorbidities [37]. Another study showed that patients with systemic sclerosis complicated by pericardial effusion and tamponade had high morbidity and mortality rates, with tamponade having greater mortality compared to pericardial effusion as mortality rates of cardiac tamponade and pericardial effusion without tamponade were 17.7% and 8.3% respectively. On the other hand, patients with pericardial effusion without tamponade were more likely to have other comorbidities such as pulmonary circulatory disease, congestive heart failure, end-stage renal disease, diabetes, and hypothyroidism [38].

H1N1 influenza A (H1N1) has been linked to pericarditis, and the H1N1 pandemic in Brazil led to the infection of more than 50000 cases with a 4% death [39]. H1N1 cardiovascular mortality was estimated to reach 83300 deaths worldwide [40]. Influenza viruses were associated with the development of cardiac diseases such as pericarditis, myocarditis, and cardiac tamponade. Influenza A was more linked to the development of these conditions compared to influenza B, and in patients having isolated pericarditis, death was not noted. However, the presence of cardiac tamponade was associated with fatality, and it was more noted in isolated pericarditis compared to myopericarditis. Cardiogenic shock was commonly associated with myopericarditis caused by influenza infection with a fatality rate of 19% [41]. Thus, the influenza virus especially the H1N1 pandemic might be linked to the gradual increase of pericardial disease mortality rates noted in our study in the period of 2000-2015.

During segment two (2016-2020), there was a notable decline in mortality rates, followed by an upturn in segment three (2020-2022). From 2016 to 2020, the decrease observed in mortality rates related to pericardial diseases can be explained by improvements in healthcare practices, including enhanced physician training in the diagnosis of pericardial diseases, increased accessibility to echocardiograms, and increased number of cardiologists [1,42-45]. Studies consistently reported that incorporating echocardiographic screening improved the prediction of major heart diseases in primary care settings [46,47]. Echocardiography plays a pivotal role in differentiating myocarditis from other conditions with similar clinical presentations, such as acute valvular heart disease, takotsubo inflammatory cardiomyopathy, and acute myocardial infarction. Additionally, it guides the performance of endomyocardial biopsies [48].

The sharp rise in segment three in both pericardial diseases and pericardial effusion mortality rates relates to the period during the COVID-19 pandemic in which healthcare was hugely directed to serving COVID-19 patients. The COVID-19 vaccines were reported to have led to pericardial diseases, specifically pericarditis. A systematic review and meta-analysis of patients who received mRNA COVID-19 vaccine compared to those who did not in the absence of COVID-19 infection showed that patients who received the vaccine showed a higher incidence of pericarditis and myocarditis [49]. Moreover, COVID-19 has been implicated in high incidences of pericardial effusion and increased mortality rate. However, a recent study reported that receipt of COVID-19 vaccines (XBB.1.5-containing vaccine) was not associated with a statistically significant increased rate of hospital contacts for any of the 28 different adverse events, including pericarditis within 28 days after vaccination [50]. In a large retrospective study, acute pericarditis was not common among patients hospitalized for COVID-19 infection. However, patients who had acute pericarditis alongside COVID-19 had a higher mortality, longer hospital stays, and were more likely to develop cardiovascular complications and acute renal failure [51].

Since hospital/bed space was reserved for COVID-19 patients, patients that had mild pericardial diseases were not hospitalized and this could have had an effect on the rise in mortality in segment three, since only severe cases with fatal outcomes sought medical services [52]. Worldwide, the COVID-19 pandemic has had an influence on many different aspects of society; however, access to healthcare for unrelated ailments has been particularly affected. The primary findings on the decrease in healthcare service utilization could be attributed to health systems giving different considerations to how they responded to the public health emergency depending on the situation [53]. Initially, efforts were primarily focused on stopping the spread of COVID-19 and giving health services the resources they required to handle the rapidly increasing demand for medical care. As a result, certain services and procedures were deemed non-essential, which resulted in a decrease in the funds allotted to meet those medical needs [54].

Pericardial diseases mortality rates based on age

The lowest mortality rates were in the 20-49 years age group; this could be explained by this group being young, healthy, and immunocompetent. Moreover, these age groups can access healthcare facilities much easier when compared to older individuals. The age groups 60-69, 70-79, and 80 years and above had mortality trends similar to those of sex differences. Starting with an increase till 2016, which might be attributed to infectious diseases and poor healthcare services [55]. 

Between 2016 and 2020, there was a decline in mortality rates, attributed to enhancements in healthcare infrastructure and the training of medical professionals [28]. However, from 2020 onward, mortality rates saw an increase, potentially linked to the impact of COVID-19 infections and the prioritization of hospital resources for COVID-19 patients. During this period, a decrease in mortality rates was noted among older age groups, particularly those aged 70-79 years compared to those aged 80 years or older. This observation may be explained by the higher prevalence of comorbidities in older age groups, which could contribute to a variety of causes of death. Consequently, the lower mortality observed in older individuals may be influenced by the presence of multiple diseases at these ages, resulting in a broader range of potential causes of death. Older patients often present with symptoms such as dyspnea, elevated systolic blood pressure, and pericardial, and pleural effusions, posing challenges in diagnosing pericarditis in this demographic group [5]. For example, elderly females with acute pericarditis may manifest dyspnea rather than chest pain, lack characteristic physical findings like rubs and fever, and exhibit nonspecific electrocardiogram findings, making the diagnosis of pericarditis difficult using established criteria [4].

Pericardial effusion mortality rates in our study showed a similar trend to overall pericardial diseases mortality. However, the mortality rates due to pericarditis did not exhibit the same pattern during 2020-2022, which was the COVID-19 pandemic period. It was discovered that pericarditis cases linked to COVID-19 were frequently complicated with significant pericardial effusions requiring pericardial drainage [56-58]. Furthermore, one study indicated that between 7% and 17% of COVID-19 patients had acute cardiac injury, and the death rate was greater in these patients [59]. This could be the reason for the decline in pericarditis-related death rates during the COVID-19 pandemic (2020-2022) compared to the rise in cases complicated with pericardial effusion. In this article, a gap was covered regarding the mortality trends of pericardial diseases in Brazil. However, a bibliometric study that analyzed the data of the top-cited publications on pericardial diseases around the world showed that countries from North America and Europe constituted the highest number of highly cited publications around the world [60]. Thus, this might suggest that pericardial diseases studies are neglected in Latin America with the need for more studies in this field.

To enhance our comprehension of pericardial diseases in Brazil, we advocate for the creation of a national registry dedicated to cataloging these conditions. Rather than lumping them together under a single umbrella term as is currently the case in Brazil, the registry should meticulously categorize each pericardial disease with its corresponding ICD code. This approach will facilitate the collection of patient data and enable the monitoring of trends, thus serving as a valuable resource for epidemiological studies, outcomes research, and the assessment of healthcare delivery models. Ultimately, such a registry has the potential to contribute to reducing mortality rates associated with pericardial diseases.

A national registry holds the potential to yield comprehensive data on various aspects such as incidence rates, demographic profiles, survival outcomes, procedural interventions, physician feedback, and assessments of healthcare quality, among other benefits [61]. By systematically collecting data, we can deepen our understanding of pericardial diseases, the demographics they affect, associated comorbidities, and their natural progression over time. This lays the groundwork for a more nuanced comprehension of causative factors and related aspects of the disease, ultimately facilitating early detection, treatment, and prevention strategies. Moreover, a well-established registry has the capacity to gather insights not only from healthcare providers but also from caregivers, family members, and other contributors involved in patient care. The wealth of information made available to researchers and healthcare institutions through such a registry empowers them to continually refine treatment approaches and advance patient outcomes. The data collected can serve as the foundation for crafting care guidelines, assisting caregivers in providing optimal patient care, and fueling quality improvement endeavors within healthcare facilities. Furthermore, researchers can harness the registry to scrutinize treatment modalities and their corresponding outcomes, as well as to devise and execute clinical trials with precision. Expedient identification and recruitment of eligible patients are paramount in the planning of clinical trials. A robust registry that centralizes patients' data plays a pivotal role in streamlining this crucial process. These data points are particularly crucial in determining the efficacy of interventions aimed at alleviating symptoms and mitigating adverse outcomes.

Limitation

Limitations of this study include its observational and retrospective design, which does not allow for a stern conclusion but raises hypotheses and awareness, vital for the evidence-based implementation of necessary political, social, and administrative measures. Similar to other developing countries, Brazil still experiences challenges regarding the quality of data collection with a number of unreported data, which might have impacted this study. Another noteworthy limitation is the process of retrieval of information from the database, with possible biases generated by data entry errors, such as the under-reported deaths or failures in the completion of the death certificate by the physicians. 

## Conclusions

This comprehensive analysis spanning two decades (2000-2022), examined the mortality trends of pericardial diseases in Brazil and revealed relative stability overall. Males exhibited an overall higher mortality number due to pericardial diseases; however, females showed the most significant increase in mortality trend throughout the whole period. In the first segment (2000-2015), mortality rose across all cohorts, which was attributed to substandard healthcare facilities and infectious diseases like TB. The second segment (2016-2020) saw a decline in mortality, likely due to improved healthcare, particularly the increased availability of echocardiograms. However, the third segment (2020-2022) witnessed a sharp rise in mortality, coinciding with the COVID-19 pandemic, with post-COVID-19 symptoms and adverse effects of COVID-19 vaccines, particularly pericarditis. Pericarditis-related death rates declined compared to pericardial effusion, and mortality rates correlated directly with age, with older cohorts experiencing higher mortality due to increased comorbidities, and decline in health and immunocompetency.

## References

[1] Khandaker MH, Espinosa RE, Nishimura RA, Sinak LJ, Hayes SN, Melduni RM, Oh JK (2010). Pericardial disease: diagnosis and management. Mayo Clin Proc.

[2] Lazarou E, Tsioufis P, Vlachopoulos C, Tsioufis C, Lazaros G (2022). Acute pericarditis: update. Curr Cardiol Rep.

[3] Chiabrando JG, Bonaventura A, Vecchié A (2020). Management of acute and recurrent pericarditis: JACC state-of-the-art review. J Am Coll Cardiol.

[4] Kytö V, Sipilä J, Rautava P (2014). Clinical profile and influences on outcomes in patients hospitalized for acute pericarditis. Circ.

[5] Lazaros G, Antonopoulos AS, Lazarou E, Vlachopoulos C, Vogiatzi G, Vassilopoulos D, Tousoulis D (2021). Age- and sex-based differences in patients with acute pericarditis. Eur J Clin Invest.

[6] Adler Y, Charron P, Imazio M (2015). 2015 ESC guidelines for the diagnosis and management of pericardial diseases. Kardiol Pol.

[7] Kumar N, Pandey A, Jain P, Garg N (2016). Acute pericarditis-associated hospitalization in the USA: a nationwide analysis, 2003-2012. Cardiol.

[8] Prepoudis A, Koechlin L, Nestelberger T (2022). Incidence, clinical presentation, management, and outcome of acute pericarditis and myopericarditis. Eur Heart J Acute Cardiovasc Care.

[9] Del Portillo-Navarrete JH, Pizano A, Benavides J, Palacio AM, Moreno-Medina K, Cabrales J, Echeverri D (2022). Unveiling the causes of pericardial effusion in a contemporary case series of pericardiocentesis in Latin America. Sci Rep.

[10] Sigvardt FL, Hansen ML, Kristensen SL (2020). Risk factors for morbidity and mortality following hospitalization for pericarditis. J Am Coll Cardiol.

[11] Mutyaba AK, Balkaran S, Cloete R, du Plessis N, Badri M, Brink J, Mayosi BM (2014). Constrictive pericarditis requiring pericardiectomy at Groote Schuur Hospital, Cape Town, South Africa: causes and perioperative outcomes in the HIV era (1990-2012). J Thorac Cardiovasc Surg.

[12] Imazio M, Gaita F, LeWinter M (2015). Evaluation and treatment of pericarditis: a systematic review. J Am Med Assoc.

[13] Benjamin SR, Mohammad A, Shankar R (2022). Does tuberculosis affect surgical outcomes following pericardiectomy for chronic constrictive pericarditis? Twelve years’ experience from a tertiary care center in India. Indian J Thorac Cardiovasc Surg.

[14] Yadav S, Shah S, Iqbal Z (2021). Pericardiectomy for constrictive tuberculous pericarditis: a systematic review and meta-analysis on the etiology, patients’ characteristics, and the outcomes. Cureus.

[15] Magalhaes T, Chalegre KD, Braga C, Foy BD (2020). The endless challenges of arboviral diseases in Brazil. Trop Med Infect Dis.

[16] (2024). Brazil starts mass vaccination amid upsurge in dengue fever. https://www.theguardian.com/world/2024/feb/07/brazil-dengue-fever-mass-vaccination.

[17] Giri A, Acharya S, Kamat S, Shukla S, Kumar S (2022). Myopericarditis - a catastrophic complication of dengue fever. J Family Med Prim Care.

[18] Imazio M, Brucato A, Maestroni S, Cumetti D, Belli R, Trinchero R, Adler Y (2011). Risk of constrictive pericarditis after acute pericarditis. Circ.

[19] Imazio M, Adler Y (2013). Management of pericardial effusion. Eur Heart J.

[20] Pratiwi I, Agustina H, Martanto E (2016). Characteristic of pericardial effusion patient based on age, gender, cytological and clinical diagnosis at SMF pathology anatomy Hasan Sadikin Bandung Hospital in 2009-2013. Ina J Chest Crit Emerg Med.

[21] Laufer-Perl M, Havakuk O, Shacham Y (2017). Sex-based differences in prevalence and clinical presentation among pericarditis and myopericarditis patients. Am J Emerg Med.

[22] Mehdizadegan N, Mohammadi H, Amoozgar H, Pournajaf S, Edraki MR, Naghshzan A, Yazdani MN (2022). Pericardial effusion among children: retrospective analysis of the etiology and short-term outcome in a referral center in the south of Iran. Health Sci Rep.

[23] Wybraniec MT, Kampka Z, Drabczyk M (2023). Clinical characteristics and risk factors of in-hospital mortality among patients undergoing percutaneous pericardiocentesis. Front Cardiovasc Med.

[24] Pennacchioni A, Nanni G, Sgura FA (2021). Percutaneous pericardiocentesis for pericardial effusion: predictors of mortality and outcomes. Intern Emerg Med.

[25] Mitiku TY, Heidenreich PA (2011). A small pericardial effusion is a marker of increased mortality. Am Heart J.

[26] Queiroz CM de, Cardoso J, Ramires F (2021). Pericardial effusion and cardiac tamponade: etiology and evolution in the contemporary era. Int J Cardiovasc Sci.

[27] Bigoni A, Malik AM, Tasca R, Carrera MB, Schiesari LM, Gambardella DD, Massuda A (2022). Brazil's health system functionality amidst of the COVID-19 pandemic: an analysis of resilience. Lancet Reg Health Am.

[28] Roman A (2023). A closer look into Brazil’s healthcare system: what can we learn?. Cureus.

[29] Little WC, Freeman GL (2006). Pericardial disease. Circ.

[30] Maisch B, Seferović PM, Ristić AD (2004). Guidelines on the diagnosis and management of pericardial diseases executive summary; the task force on the diagnosis and management of pericardial diseases of the European Society of Cardiology. Eur Heart J.

[31] Imazio M, Demichelis B, Cecchi E, Belli R, Ghisio A, Bobbio M, Trinchero R (2003). Cardiac troponin i in acute pericarditis. J Am Coll Cardiol.

[32] Diaconu R, Donoiu I, Mirea O, Bălşeanu TA (2021). Testosterone, cardiomyopathies, and heart failure: a narrative review. Asian J Androl.

[33] Kruk ME, Gage AD, Joseph NT, Danaei G, García-Saisó S, Salomon JA (2018). Mortality due to low-quality health systems in the universal health coverage era: a systematic analysis of amenable deaths in 137 countries. Lancet.

[34] Mayosi BM, Wiysonge CS, Ntsekhe M (2008). Mortality in patients treated for tuberculous pericarditis in sub-Saharan Africa. S Afr Med J.

[35] Pankuweit S, Ristić AD, Seferović PM, Maisch B (2005). Bacterial pericarditis: diagnosis and management. Am J Cardiovasc Drugs.

[36] Ganji M, Ruiz J, Kogler W, Lung J, Hernandez J, Isache C (2019). Methicillin-resistant Staphylococcus aureus pericarditis causing cardiac tamponade. IDCases.

[37] Chen Z, Chu M, Liu L (2022). Genetic prion diseases presenting as frontotemporal dementia: clinical features and diagnostic challenge. Alzheimers Res Ther.

[38] Basyal B, Ullah W, Derk CT (2023). Pericardial effusions and cardiac tamponade in hospitalized systemic sclerosis patients: analysis of the national inpatient sample. BMC Rheumatol.

[39] Cândido EL, Costa MS, Junior JG, Moreira MRC (2020). Influenza A/H1N1 e Covid-19 no Brasil: impactos e diferenças epidemiológicas. Rev Epidemiol Controle Infecção.

[40] Dawood FS, Iuliano AD, Reed C (2012). Estimated global mortality associated with the first 12 months of 2009 pandemic influenza A H1N1 virus circulation: a modelling study. Lancet Infect Dis.

[41] Radovanovic M, Petrovic M, Barsoum MK (2022). Influenza myopericarditis and pericarditis: a literature review. J Clin Med.

[42] Lenarduzzi Júnior RM, de Almeida Neto OP, Pedrosa LA, Silva PC, Coelho VM, Resende ES, Mendes DS (2021). Electrocardiographic and echocardiographic profile of patients with heart failure. Am J Cardiovasc Dis.

[43] Chamsi-Pasha MA, Sengupta PP, Zoghbi WA (2017). Handheld echocardiography: current state and future perspectives. Circ.

[44] Lee J, Chen T, Gill E (2022). Interventional echocardiography: opportunities and challenges in an emerging field. Echocardiogr.

[45] Ribas S (2023). Medical Demography in Brazil 2023 [Book in Portuguese]. https://amb.org.br/wp-content/uploads/2023/02/DemografiaMedica2023_8fev-1.pdf.

[46] Diamantino AC, Nascimento BR, Nunes MC (2021). Impact of incorporating echocardiographic screening into a clinical prediction model to optimise utilisation of echocardiography in primary care. Int J Clin Pract.

[47] Nascimento BR, Beaton AZ, Nunes MC (2019). Integration of echocardiographic screening by non-physicians with remote reading in primary care. Heart.

[48] Simão AF, Precoma DB, Andrade JP (2013). I Brazilian guidelines for cardiovascular prevention [Article in Portuguese]. Arq Bras Cardiol.

[49] Alami A, Krewski D, Farhat N (2023). Risk of myocarditis and pericarditis in mRNA COVID-19-vaccinated and unvaccinated populations: a systematic review and meta-analysis. BMJ Open.

[50] Andersson NW, Thiesson EM, Hviid A (2024). Adverse events after XBB.1.5-containing COVID-19 mRNA vaccines. J Assoc Am Med Coll.

[51] Li P, Shi A, Lu X (2023). Incidence and impact of acute pericarditis in hospitalized patients with COVID-19. J Am Heart Assoc.

[52] Alves L (2021). Brazilian ICUs short of drugs and beds amid COVID-19 surge. Lancet.

[53] (2024). Pulse survey on continuity of essential health services during the COVID-19 pandemic: interim report. https://www.who.int/publications/i/item/WHO-2019-nCoV-EHS_continuity-survey-2020.1.

[54] Ma X, Vervoort D, Reddy CL, Park KB, Makasa E (2020). Emergency and essential surgical healthcare services during COVID-19 in low- and middle-income countries: a perspective. Int J Surg.

[55] Walicka M, Puzianowska-Kuznicka M, Chlebus M (2021). Relationship between age and in-hospital mortality during 15,345,025 non-surgical hospitalizations. Arch Med Sci.

[56] Amoozgar B, Kaushal V, Mubashar U, Sen S, Yousaf S, Yotsuya M (2020). Symptomatic pericardial effusion in the setting of asymptomatic COVID-19 infection: a case report. Medicine (Baltimore).

[57] Ortiz-Martínez Y, Cabeza-Ruiz LD, Vásquez-Lozano SH, Villamil-Gómez WE, Rodriguez-Morales AJ (2020). Pericarditis in a young internal medicine resident with COVID-19 in Colombia. Travel Med Infect Dis.

[58] Asif T, Kassab K, Iskander F, Alyousef T (2020). Acute pericarditis and cardiac tamponade in a patient with COVID-19: a therapeutic challenge. Eur J Case Rep Intern Med.

[59] Shi S, Qin M, Shen B (2020). Association of cardiac injury with mortality in hospitalized patients with COVID-19 in Wuhan, China. JAMA Cardiol.

[60] Jain V, Gupta K, Eken HN (2023). Current status of global research on pericardial diseases: a bibliometric analysis of the top 100 from Web of Science. Arch Med Sci Atheroscler Dis.

[61] Meltzer SN, Weintraub WS (2020). The role of national registries in improving quality of care and outcomes for cardiovascular disease. Methodist Debakey Cardiovasc J.

